# Identifying individuals with chronic pain after knee replacement: a population-cohort, cluster-analysis of Oxford knee scores in 128,145 patients from the English National Health Service

**DOI:** 10.1186/s12891-018-2270-9

**Published:** 2018-10-02

**Authors:** Rafael Pinedo-Villanueva, Sara Khalid, Vikki Wylde, Rachael Gooberman-Hill, Anushka Soni, Andrew Judge

**Affiliations:** 1 Nuffield Department of Orthopaedics, Rheumatology and Musculoskeletal Sciences, University of Oxford, Botnar Research Centre, Windmill Road, Headington, Oxford, OX3 7LD UK; 2 MRC Lifecourse Epidemiology Unit, University of Southampton, Southampton, UK; 3 Musculoskeletal Research Unit, Translational Health Sciences, Bristol Medical School, University of Bristol, Learning and Research Building, Level 1, Southmead Hospital, Bristol, BS10 5NB UK; 4 Wellcome Centre for Integrative Neuroimaging, FMRIB, Nuffield Department of Clinical Neurosciences, University of Oxford, Oxford, UK; 5 National Institute for Health Research Bristol Biomedical Research Centre, University Hospitals Bristol NHS Foundation Trust and University of Bristol, Bristol, UK

**Keywords:** Chronic pain, Knee replacement, Cluster-analysis, Oxford knee score, Observational study, NHS England

## Abstract

**Background:**

Approximately one in five patients undergoing knee replacement surgery experience chronic pain after their operation, which can negatively impact on their quality of life. In order to develop and evaluate interventions to improve the management of chronic post-surgical pain, we aimed to derive a cut-off point in the Oxford Knee Score pain subscale to identify patients with chronic pain following knee replacement, and to characterise these patients using self-reported outcomes.

**Methods:**

Data from the English Patient-Reported Outcome Measures (PROMs) programme were used. This comprised patient-reported data from 128,145 patients who underwent primary knee replacement surgery in England between 2012 and 2015. Cluster analysis was applied to derive a cut-off point on the pain subscale of the Oxford Knee Score.

**Results:**

A high-pain group was identified, described by a maximum of 14 points in the Oxford Knee Score pain subscale six months after surgery. The high-pain group, comprising 15% of the sample, was characterised by severe and frequent problems in all pain dimensions, particularly in pain severity, night pain and limping, as well as in all dimensions of health-related quality of life.

**Conclusions:**

Patients with Oxford Knee Score pain subscale scores of 14 or less at six months after knee replacement can be considered to be in chronic pain that is likely to negatively affect their quality of life. This derived cut-off can be used for patient selection in research settings to design and assess interventions that support patients in their management of chronic post-surgical pain.

**Electronic supplementary material:**

The online version of this article (10.1186/s12891-018-2270-9) contains supplementary material, which is available to authorized users.

## Background

Pain and functional limitations due to knee osteoarthritis (OA) is a leading health concern and a major global contributor to years lived with disability [1]. For people living with advanced stages of the disease, knee replacement (KR) can be performed to provide pain relief, restore function, and improve quality of life. However, chronic pain after KR is common: approximately 20% of patients report moderate or severe pain at between 3 months and 5 years after surgery [2].

With approximately 700,000 KR performed per year in the US [3] and 108,000 performed during 2016 in England, Wales, Northern Ireland and the Isle of Man [4], large numbers of people are living with chronic pain after KR. These patients experience high levels of pain-related distress [5] and reduced ability to participate in work, family life and valued social activities, which may contribute to social isolation [6].

Often patients do not seek care for chronic pain [7]. The ability to characterise and identify people with chronic pain following KR is a necessary first step to developing, evaluating, and implementing interventions which aim to improve the management of this condition. Although cut-off points on a visual analogue scale have been suggested to distinguish between mild, moderate and severe general pain [8], no standard cut-off points are available for patients following a KR. Identifying individuals with chronic pain early during their post-operative recovery will facilitate the delivery of timely and targeted interventions to improve pain outcomes. This requires a robust and standardised method for identifying people in chronic pain following KR that can be used across a range of research settings.

The aim of this study was to identify a cut-off point on the pain subscale of the commonly-used Oxford Knee Score (OKS) that can be used to identify patients with high levels of pain 6 months after KR.

## Methods

### Study design

Data were obtained from the patient-level, anonymized English NHS Patient-Reported Outcome Measures (PROMs) programme available from the NHS Digital website [9]. All patients undergoing KR through the English NHS, or on behalf of it, are eligible to be included in the programme. Two questionnaires are sent to participants before and after surgery by the PROMs programme, a disease-specific and a generic quality of life measure: the OKS [10] and the EQ-5D [11]. For this analysis, data were extracted for UK financial years 2012 through 2015.

### Participants

Patients undergoing primary KR, with complete post-operative OKS were included in the present study. There were no restrictions on age or sex. The dataset did not include the indication for or type of surgery performed, hence records were included regardless of the underlying condition or type of KR. As the unit of analysis was KRs and records were fully anonymised, it was not possible to determine if patients had their contralateral knee replaced within the same period of analysis. It was therefore possible for patients to be included twice in the dataset.

### Data and outcome measure

The OKS is a 12-item score which measures knee pain, stiffness, and functional disability within the previous 4 weeks [10]. Responses are scored using a 0–4 Likert scale. Item scores are summed to calculate a total score ranging from 0 (worst possible score) to 48 (best possible score). The PROMS programme collects post-operative data 6 months after surgery. Chronic post-surgical pain is widely accepted to be pain of at least three to 6 months duration that develops or increases in intensity after a surgical procedure and significantly affects health-related quality of life [12, 13]. This is applicable to KR, as most pain relief is obtained in the first three to 6 months [14, 15] and continuing pain at 6 months after surgery is a cause of dissatisfaction [16].

Previous work has identified pain- and function-related subscales within the OKS [17], with further details given in Additional file 1. A raw OKS pain subscale (OKS-PS) summary score can be calculated by summing the responses of its seven items, hence ranging from 0 (most pain) to 28 (least pain). Although the OKS, and particularly the pain subscale, could be considered an unidimensional measure of highly-correlated items, each item captures a different dimension of pain. Some pain items are highly intertwined with function, reflected by the inclusion of two items in both the pain and function components [17]. Our analysis allowed for each item to independently contribute to the generation of pain severity clusters.

### Statistical methods

To identify the high-pain group, hierarchical clustering was used to group patients into clusters based on the similarity of their OKS-PS item scores. Hierarchical clustering is commonly used, easily interpretable and has been applied in several previous studies on knee pain in order to identify pain profiles, and broader subgroups which are then examined for correlation with pain [18–21]. Cluster analysis splits a set of participants into groups or clusters, so that participants within the same cluster are most similar, and any two clusters are as distinct as possible from one another. Agglomerative hierarchical clustering follows a sequence whereby clusters are repetitively merged until a pre-specified number of clusters is achieved, or all participants have been merged into one single cluster. We conducted agglomerative clustering with all patients beginning as individual clusters, which were then successively merged into all possible numbers of clusters, starting at two (the natural minimum) up to 28 clusters (the maximum, where each cluster would correspond to each possible value of the OKS-PS). This constituted the essence of our data-driven approach, considering all possibilities allowed by the data instead of setting discretionary limits on the number of clusters to be identified. We assumed that it is not known, a-priori, if subgroups of KR patients exist based on their levels of pain and, if so, how many there are. By applying a data-driven approach, we allowed the model to answer these questions based on the features of our population.

For each set of clusters (2 to 28), the distribution of the OKS-PS corresponding to the highest-pain cluster, i.e. that reporting the lowest values, was examined. This was used to derive a cut-off point, defined as the highest value taken by the highest-pain cluster on the OKS-PS. Changes in the cut-off point as the number of clusters varied are reported. Further details on the methodology have been published previously [22].

Uncertainty around the cut-off point was measured undertaking a secondary analysis which repeated the hierarchical clustering on 100 random re-orderings of the sample, and by additionally applying k-means clustering, another commonly used method which adds an additional level of variability as cluster initialisation is randomly assigned in every iteration. To assess the choice of the cut-off point, sensitivity and specificity were calculated considering ‘true cases’ those patients included in the highest–pain cluster from the primary analysis at the lowest number of clusters (*k*) at which the cut-off was identified. These measures are useful because the analysis produced clusters based on answers to seven different items, hence clusters were likely to report overlapping overall OKS-PS scores. This secondary analysis was also conducted as an internal validation by exploring an alternative approach to generating cut-off points using the same data, although differently ordered.

Based on the cut-off point identified in the primary analysis, patients were placed into either a ‘high-pain’ or a ‘low-pain’ group, and scores reported for the overall OKS, OKS subscales, OKS-PS items, EQ-5D Visual Analogue Scale (VAS), and the EQ-5D summary score comprising mobility, self-care, usual activities, pain/discomfort, and anxiety/depression. EQ-5D dimensions are scored using a 1–3 Likert scale [11] and a summary index calculated by applying UK general population preference weights [23]. The summary score is anchored at 0 signifying ‘death’ and 1 ‘perfect health’, negative values being possible and interpreted as health states worse than death. Answers to two questions about the results and impact of the surgery are also reported by group. Analyses were performed using Matlab R2015 and Stata.

## Results

During the study period, 128,145 records from primary KR patients were reported in the PROMs dataset with returned post-operative questionnaires. Of these, 2081 (1.6%) records were excluded as they were missing ≥1 answers for the OKS-PS items. Therefore, 126,064 records were used for analysis, of which 54% were from females and 73% from patients aged 60–79 years. Table 1 shows participants’ demographic characteristics.

**Table 1:** Cohort description

Observations: n	126,064
Gender: n (%)	
Female	68,114 (54%)
Male	49,306 (39%)
Not specified	106 (0%)
Missing	8538 (7%)
Age band: n (%)	
40–49 years of age	258 (0%)
50–59 years of age	11,380 (9%)
60–69 years of age	43,583 (35%)
70–79 years of age	48,073 (38%)
80–89 years of age	14,226 (11%)
90+ years of age	6 (0%)
Missing	8538 (7%)
Pre-op OKS score^1^	
Mean (SD)	19.0 (8)
Median (IQR)	19 (11)
Missing	1473 (1%)
Pre-op EQ-5D index^2^	
Mean (SD)	0.414(0.31)
Median (IQR)	0.587 (0.603)
Missing	7077 (6%)
Pre-op EQ-5D VAS^3^ “Your own health state today”	
Mean (SD)	68.2(20)
Median (IQR)	70 (25)
Missing	13,110 (10%)
“How would you describe the results of your operation?” n (%)	
Excellent	31,827 (25%)
Very good	44,472 (35%)
Good	30,670 (24%)
Fair or Poor	18,355 (15%)
Missing	740 (1%)
“Overall, how are your problems now, compared to before your operation?” n (%)	
Much better	92,164 (73%)
A little better	20,276 (16%)
About the same	5775 (5%)
A little or much worse	7388 (6%)
Missing	461 (0%)
Post-op OKS score^a^	
Mean (SD)	35.2 (10)
Median (IQR)	37 (14)
Missing	46 (0%)
Post-op EQ-5D index^b^	
Mean (SD)	0.734(0.25)
Median(IQR)	0.760 (0.344)
Missing	6084 (5%)
Post-op EQ-5D VAS^c^ “Your own health state today”	
Mean (SD)	73.8 (20)
Median (IQR)	80 (25)
Missing	6746 (5%)

^a^captures knee pain and function, scored from 0 to 48 where 0 indicates most and 48 least pain and functional limitations

^b^a health-related quality of life measure where 0 represents death and 1 refers to perfect health

^c^a Visual Analogue Scale ranging from 0 to 100, where 0 represents the worst and 100 the best imaginable health state

### Identification of a cut-off point

**Fig. 1:**
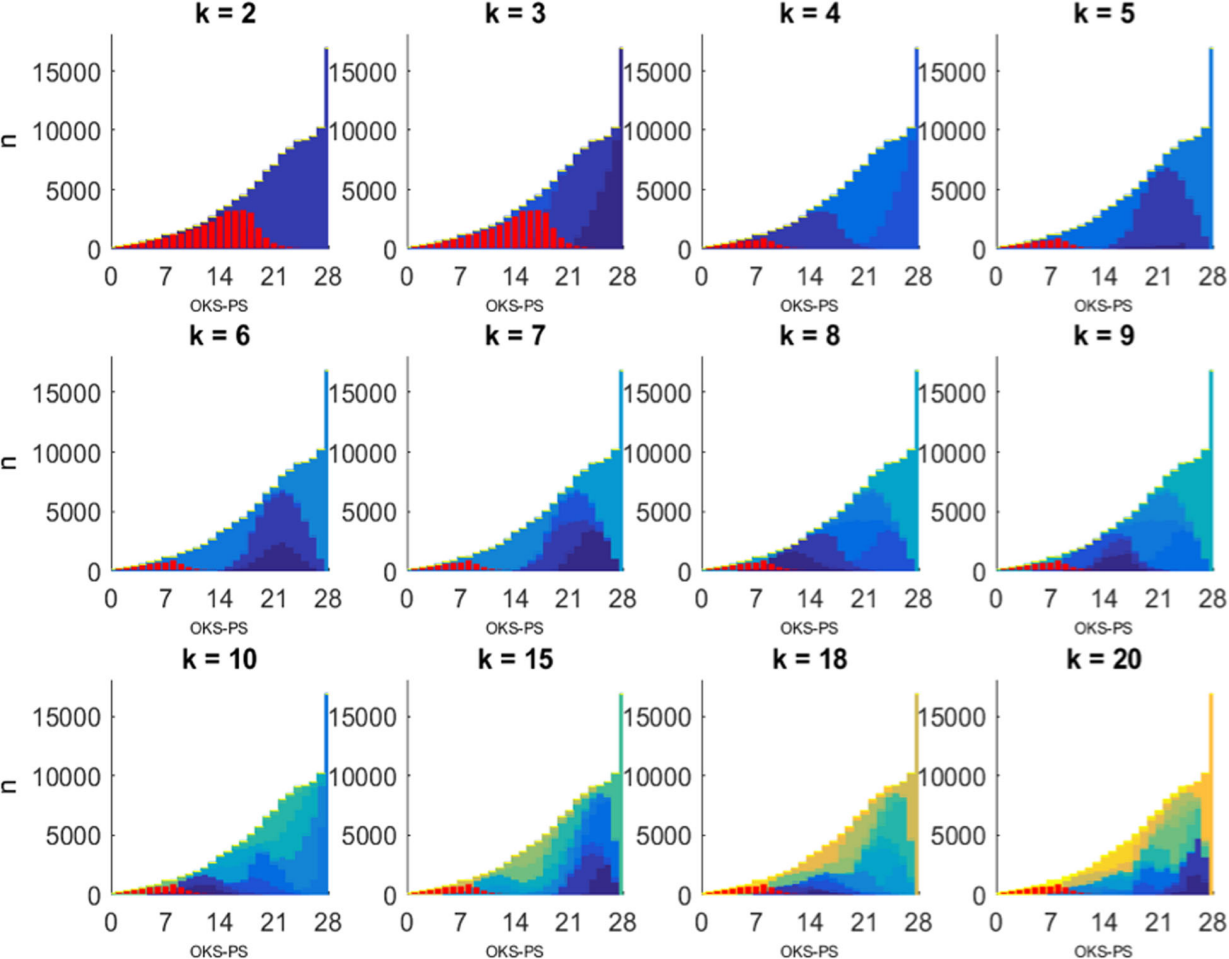
Distribution of clusters over OKS-PS score. Corresponds to primary analysis. k denotes the number of clusters derived. Y-axis represents the number of patients (n). Each cluster is shown in a different colour. The left-most cluster on the OKS-PS scale, i.e. the high pain-cluster, is highlighted in red

**Fig. 2:**
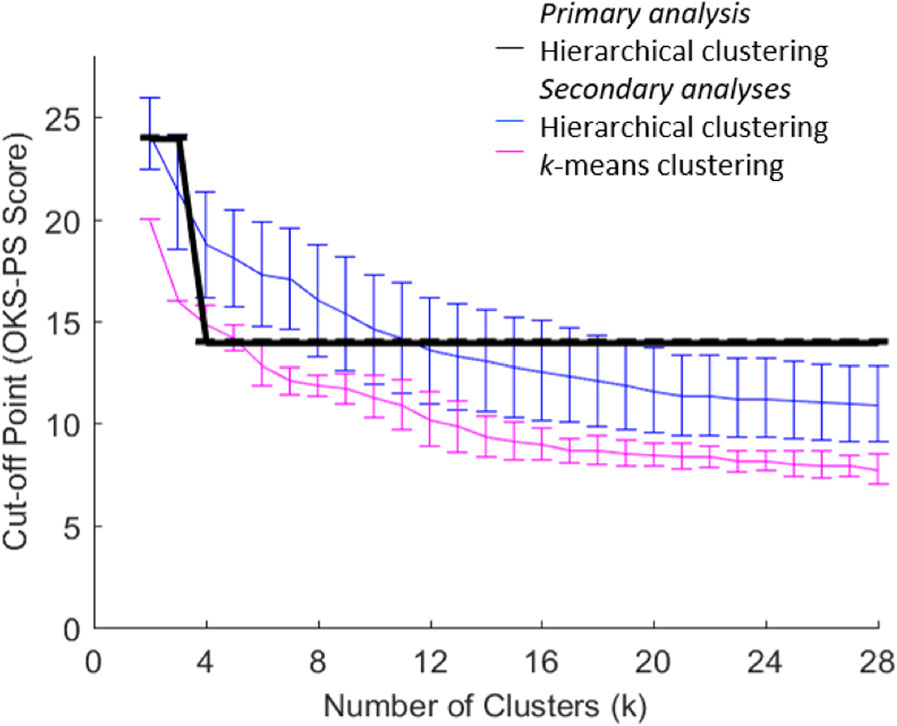
OKS-PS cut-off point over number of clusters. Cut-off point corresponding to the upper limit of the OKS-PS distribution of the high-pain cluster. Results from the primary analysis are shown in black, obtained by applying hierarchical clustering to the original ordering of the sample. Results from the secondary analyses are shown in blue and pink, reporting ±1sd at either side of the average cut-off points derived after 100 random re-orderings of the sample. Results in blue correspond to hierarchical clustering and pink to k-means clustering

Figure 1 shows the distribution of different-size clusters over the OKS-PS summary score from the primary analysis. When the clustering algorithm derives *k* = 2 clusters, the OKS-PS distributions for the two clusters have a large overlap. As the number of derived clusters increases, the degree of overlap in their corresponding OKS-PS distributions reduces and both the mode and the upper limit shift. Figure 2 illustrates this shift in the resulting cut-off point, in particular how it decreases as the number of clusters increases from *k* = 2 to *k* = 4. However, for *k* ≥ 4, the cut-off point remains constant at OKS-PS = 14, implying that the OKS-PS score range of 0–14 characterises the high-pain cluster whether the sample is split into four or more clusters. Using OKS-PS ≤ 14 to identify participants in chronic pain led to a sensitivity of 100% and specificity of 89%.

The cut-off points obtained through secondary analysis were different to that obtained through the primary analysis. As Fig. 2 shows, running 100 random re-orderings and applying hierarchical clustering led to a mean cut-off point at k = 4 clusters of OKS-PS = 18.5 (SD = 2.6), whilst when *k*-means was applied the mean cut-off was OKS-PS = 14.7 (SD = 0.9). The Figure also illustrates that these cut-off points are not constant with increasing numbers of clusters*.* At OKS-PS = 18.5, sensitivity and specificity were 100% and 74%, whereas at OKS-PS = 14.7 they were 100% and 89%, respectively.

### Characterisation of the high-pain group

Using the OKS-PS cut-off of 14, a total of 18,522 out of 126,064 (14.7%) patients with KR were found to be in the high-pain group. Table 2 shows the demographics and outcome scores for the high- and low-pain groups.

**Table 2:** Cohort description by pain group

	Low pain (OKS-PS > 14)	High pain (OKS-PS ≤ 14)
Observations: n (%)	107,542 (85.3%)	18,522 (14.7%)
Gender: n (%)		
Female	57,771 (54%)	10,343 (56%)
Male	42,683 (40%)	6623 (36%)
Not specified	98 (0%)	8 (0%)
*Missing*	*6990 (7%)*	*1548 (8%)*
Age band: n (%)		
40–49 years of age	167 (0%)	91 (0%)
50–59 years of age	8668 (8%)	2712 (15%)
60–69 years of age	37,112 (35%)	6471 (35%)
70–79 years of age	42,070 (39%)	6003 (32%)
80–89 years of age	12,529 (12%)	1697 (9%)
90+ years of age	6 (0%)	0 (0%)
*Missing*	*6990 (7%)*	*1548 (8%)*
Pre-op OKS score^1^		
Mean (SD)	19.8 (8)	14.4 (8)
Median (IQR)	20 (11)	14 (10)
*Missing*	*1229 (1%)*	*244 (1%)*
Standardised pre-op OKS-Pain score ^2^		
Mean (SD)	36.5 (16)	25.6 (15)
Median (IQR)	35.7 (21.42)	25.0 (21.42)
*Missing*	*1227 (1%)*	*243 (1%)*
Standardised pre-op OKS-Function score ^3^		
Mean (SD)	48.0 (18)	36.4 (17)
Median (IQR)	45 (25)	35 (20)
*Missing*	*1185 (1%)*	*240 (1%)*
Pre-op EQ-5D index^4^		
Mean (SD)	0.442 (0.30)	0.249 (0.32)
Median (IQR)	0.620 (0.532)	0.159 (0.603)
*Missing*	*5887 (5%)*	*1990 (11%)*
Pre-op EQ-5D VAS^5^ “Your own health state today”		
Mean (SD)	69.6 (19)	59.8 (22)
Median (IQR)	74 (25)	60 (35)
*Missing*	*10,807 (10%)*	*2304 (12%)*
“How would you describe the results of your operation?” n (%)		
Excellent	31,212 (29%)	615 (3%)
Very good	42,716 (40%)	1756 (9%)
Good	25,755 (24%)	4915 (27%)
Fair	6740 (6%)	7273 (39%)
Poor	602 (1%)	3740 (20%)
*Missing*	*517 (0%)*	*223 (1%)*
“Overall, how are your problems now, compared to before your operation?” n (%)		
Much better	89,210 (83%)	2954 (16%)
A little better	14,271 (13%)	6005 (32%)
About the same	2286 (2%)	3489 (19%)
A little worse	1237 (1%)	3227 (18%)
Much worse	227 (0%)	2647 (14%)
*Missing*	*331 (0%)*	*150 (1%)*
Post-op OKS score^a^		
Mean (SD)	38.2 (7)	17.8 (6)
Median (IQR)	39 (11)	19 (8)
*Missing*	44 (0%)	2 (0%)
Standardised post-op OKS-Pain score^b^		
Mean (SD)	82.4 (14)	36.0 (12)
Median (IQR)	85.7 (25.0)	39.3 (17.9)
*Missing*	*0 (0%)*	*0 (0%)*
Standardised post-op OKS-Function score^c^		
Mean (SD)	75.4 (8)	38.8 (8)
Median (IQR)	80 (25)	40 (20)
*Missing*	*44 (0%)*	*2 (0%)*
Post-op EQ-5D index^d^		
Mean (SD)	0.792 (0.19)	0.395 (0.30)
Median (IQR)	0.796 (0.309)	0.516 (0.532)
*Missing*	*4934 (5%)*	*1150 (6%)*
Post-op EQ-5D VAS^e^ “Your own health state today”		
Mean (SD)	76.9 (16)	55.1 (20)
Median (IQR)	80 (20)	55 (30)
*Missing*	*5413 (5%)*	*1333 (7%)*

^a^captures knee pain and function, scored from 0 to 48 where 0 indicates most and 48 least pain and functional limitations

^b^OKS Pain subscale as captured by seven questions, scored 0–100 where 0 indicates most and 100 least pain

^c^OKS Function subscale as captured by five questions, scored 0–100 where 0 indicates most and 100 least functional limitations

^d^a health-related quality of life measure where 0 represents death and 1 refers to perfect health

^e^a Visual Analogue Scale ranging from 0 to 100, where 0 represents the worst and 100 the best imaginable health state

**Fig. 3:**
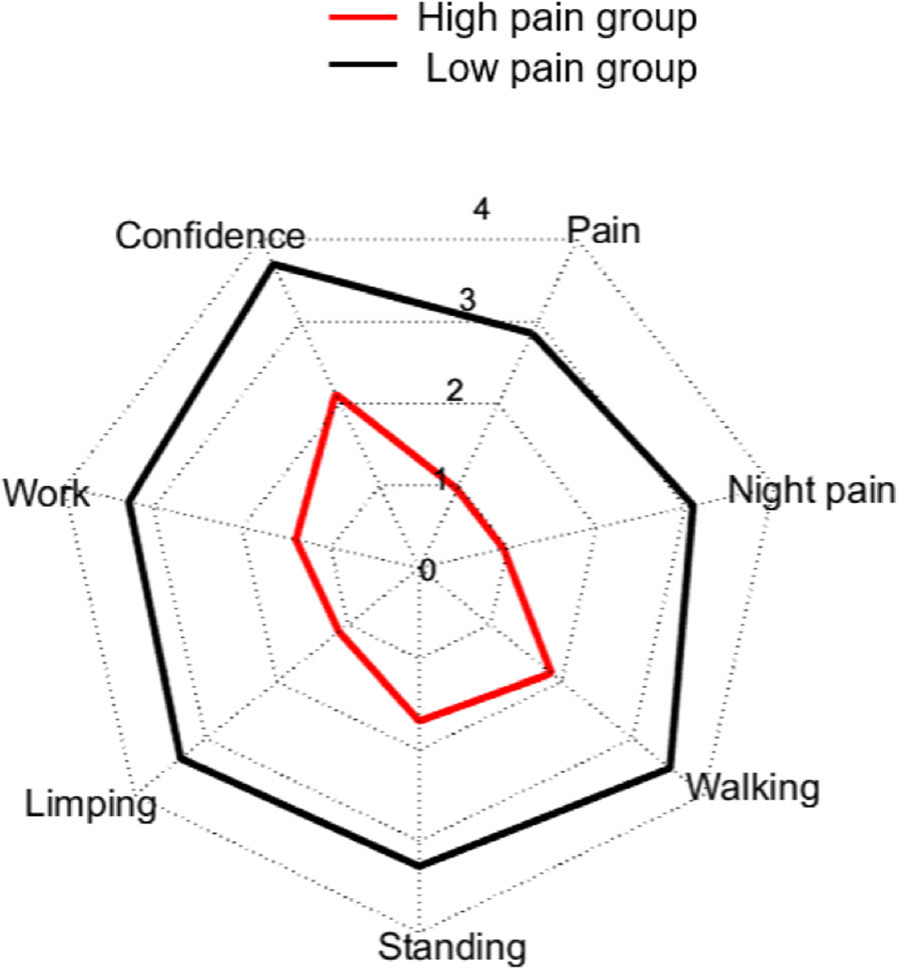
Mean OKS-PS item scores by pain group. Based on cut-off point of 14. Score of 0 means ‘most pain’ and 4 ‘least pain’ in terms of severity/frequency/impact

**Fig. 4:**
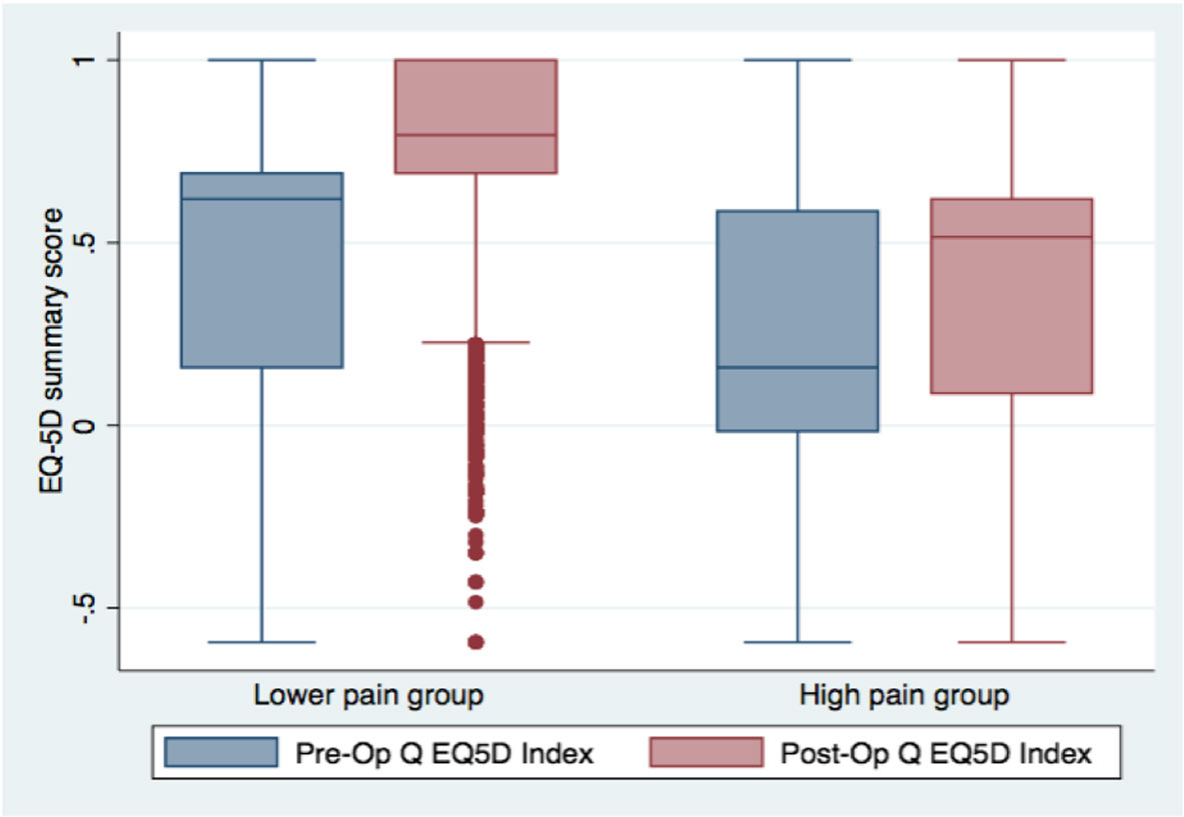
Pre- and post-operative EQ-5D summary score by pain groups

Age and sex were largely similar between both groups, with the exception of a larger proportion of 50–59 year olds and lower proportion of 70–79 year olds in the high-pain compared to the lower-pain group. Pre-operatively, patients in the high-pain group reported worse pain, function and health-related quality of life. Post-operatively, outcomes were also poorer for the high-pain group. A higher percentage of the high-pain group judged the outcome of their operation as ‘fair’ or ‘poor’, and perceived little or no improvement in their symptoms from their pre-operative status. Figure 3 shows how the high-pain group consistently reported greater pain, in average, in all OKS-PS items. The greatest differences were reported in pain severity, limping and night pain. The overall improvement of the standardised OKS-PS (scale of 0–100) was 46 for the lower-pain group and 10 for the high-pain group. Outcomes in function were even worse for the high-pain group, with a mean improvement of 2 points compared to 27 for the lower-pain group. Post-operative health-related quality of life was also worse in the high-pain group, with lower scores by approximately the same magnitude across all five dimensions. As shown in Fig. 4, however, the median of the EQ-5D summary score for the high-pain group saw a larger improvement after surgery than the lower-pain group, but the middle half of the high-pain group remained essentially within the same range of scores reported pre-operatively. Notably, 17% of the high-pain group reported post-operative EQ-5D summary scores lower than zero, compared to less than 1% for the lower-pain group.

## Discussion

Using data from a large cohort of patients in England, we have developed a standardised and robust method to identify people with chronic pain following primary KR. Clusters of patients were produced according to their responses to pain-related questions in the OKS. A specific group with the lowest OKS-PS, indicating high pain, was identified. Characterisation of this group showed an association with poor health-related quality of life. A cut-off was derived such that a patient with an OKS-PS score ≤ 14 6 months after KR could be considered to be in chronic pain which is likely to have a negative impact on health-related quality of life.

Our secondary analysis using hierarchical clustering and based on repetitive sampling with different ordering showed that the cut-off could be higher than 14. Differences between the primary and secondary analysis were due to the random data reordering in the latter and the high probability of ties during the clustering process. Ties are highly likely when the clustering variables are discrete and can only take a few values, such as in this case the OKS items which can only take the values 0 to 4. Each iteration of the clustering analysis produced different clusters as it faced ties in a different order every time, with this variation increased by the fact that the k-means method starts the clustering from random points every time. By conducting both analyses, we are able to provide a reference point for the cut-off value as well as a measure of its variability when the data and cluster initialisation are allowed to change randomly.

This cut-off point corresponds to the highest value in the OKS-PS for a participant in the high-pain group when four groups or clusters had been identified. This number of clusters is consistent with the number identified by previous studies also using cluster analysis. Egsgaard et al. identified four knee pain profiles based on biochemical and pain biomarkers, physical impairments, and psychological factors [20], whilst Frey-Law identified five distinct pain sensitivity profiles [21].

Identifying patients with chronic pain after KR revealed marked differences between the high- and lower- pain groups not only 6 months after the operation but also pre-operatively and with regards to patients’ assessment of their outcomes. Expectedly, all measures of post-operative pain and health-related quality of life were worse for patients in the chronic-pain group. They reported significantly worse scores than their counterparts in all items of the OKS-PS items as well as in all health-related quality of life dimensions. Patients with chronic pain were characterised by high pain intensity and frequency, with severity, night pain, and limping as the most serious problems. Regarding health-related quality of life, those in chronic pain reported least problems in self-care and anxiety/depression but were still notably worse than the low pain-group in all dimensions of health-related quality of life. After the operation, one in six individuals with chronic pain reported scores considered to be worse than death.

There were some difficulties in applying the cut-off on the OKS-PS to identify patients in the highest-pain cluster. Specificity of 89% revealed that some of those scoring ≤14 in their OKS-PS were not in this highest-pain cluster but in others with likely similar characteristics. The heterogeneity of outcomes after surgery for the group scoring ≤14 reflects this. Despite function remaining essentially unchanged, 32% of patients reporting their problems being worse after surgery and 60% perceiving their operation either “fair” or “poor”, in average their pain improved and 48% indicated that their problems had also improved after surgery. Therefore, this high-pain group did much worse than their counterparts, but many of them reported some improvement after surgery.

This problem could potentially be attenuated if several cut-off points based on various patient characteristics were identified. Such an approach has been used previously to identify thresholds for satisfaction after joint replacement, stratified by subgroups of baseline or change scores [24]. The authors found slight variation in the thresholds, although there was also great overlap between them. However, the groups were arbitrarily defined. We employed an data-driven approach, free from researcher intervention in the definition of groups or the number of clusters. There is no evidence to suggest that multiple cut-off points, for arbitrarily defined patient subgroups, would lead to more efficient classifications, but they would add a level of bias and make their potential application in clinical practice more complex.

Conducting a complete-case analysis was one limitation of this study. Results may therefore be affected as patients who did not complete all items of the OKS-PS may be different to those who did. However, only 1.6% of all patients in the dataset were excluded for incomplete data, thereby making any such bias unlikely. Large population cohorts such as the one used here also often suffer from loss to follow-up. In our study, however, only two patients who completed a pre-operative form did not complete the follow-up questionnaire, suggesting the data are representative of the English population.

Hierarchical cluster analysis has been used in a number of OA patient studies [18–21]. All of these studies used visual inspection of the dendogram (clustering diagram) in samples of 64 to 346 patients to identify the optimal number of clusters. Visual inspection is only practical in studies with small sample sizes. In this study, we applied data-driven clustering methods to a sample of over 120,000 KR patients and examined the highest OKS-PS value taken by the high-pain cluster without any discretionary framework imposed on the analysis. This is the first time, to our knowledge, that hierarchical clustering has been applied to large, real-world evidence data to identify groups based on their levels of chronic post-surgical pain.

The lack of a gold standard or other reference point for chronic pain following KR did not allow for a formal external validation of our method using a split sample of our data. However, our secondary analysis served as an internal validation as it evaluated the method by adding a second clustering approach whilst generating random orderings of the data. Our primary result of 14 fell within the ranges of values obtained from either method for all numbers of clusters between four and 19. In order to assess the generalisability of the cut-off point we identified as a screening tool for people with chronic pain following KR, a validation study using an external cohort is recommended.

Using the cut-off point of 14 in the OKS-PS identified in the primary analysis, 15% of primary KR patients in England between 2012 and 2015 had chronic pain 6 months after their operation. This result is consistent with previous findings: a systematic review [2] found that 10–34% of patients reported moderate to severe chronic pain after KR. In one of the UK studies included in the review, 15% of TKR patients had severe to extreme pain three to 4 years after KR [25].

## Conclusion

This study developed a cut-off score on a commonly used PROM to provide researchers with a standardised and validated approach to the identification of patients with chronic pain after TKR. We used a population-based observational cohort to identify a cut-off point in the Oxford Knee Score pain subscale of 14 at 6 months after KR which can be useful to select patients with chronic pain. Our secondary analyses indicated that the cut-off can be higher than 14. This cut-off point will facilitate the inclusion of a targeted group in clinical trials to help investigate pain characteristics, biological mechanism and evaluate interventions designed to improve support and management for people with KR who experience chronic pain. Given the growing number of KR operations being performed and the negative association found between chronic post-surgical pain and health-related quality of life, targeted interventions are likely to benefit many people. Identifying these individuals using a simple and commonly used questionnaire such as the OKS will also allow for the conduct of future qualitative studies to assess how the profile described here matches individuals’ experiences, outcomes and expectations in other settings before broader uptake in clinical practice is considered.

## Additional file

Additional file 1: Pain component subscale items of the Oxford Knee Score. Description of the items from the Oxford Knee Score included in the Pain component subscale showing their respective scoring categories. (DOCX 13 kb)

## Abbreviations

EQ-5D: Euro-Quol 5-dimension health-related quality of life questionnaire
KR: Knee replacement
NHS: National Health Service in England
NIHR: National Institute for Health Research
OA: Osteoarthritis
OKS: Oxford Knee Score
OKS-PS: Oxford Knee Score pain subscale
PROMs: Patient-Reported Outcome Measures
VAS: Visual Analogue Scale
