# Supplementary material for: Identifying individuals with chronic pain after knee replacement: a population-cohort, cluster-analysis of Oxford knee scores in 128,145 patients from the English National Health Service
Source: BMC Musculoskelet Disord. 2018 Oct 2;19:354. doi: 10.1186/s12891-018-2270-9 (PMC6169112; doi:10.1186/s12891-018-2270-9)
Supplement: Supplementary file 1 — Pain component subscale items of the Oxford Knee Score. Description of the items from the Oxford Knee Score included in the Pain component subscale showing their respective scoring categories. (DOCX 13 kb) [file 12891_2018_2270_MOESM1_ESM.docx]

| **Item label** | **Item Description** | **Scoring categories** |
| --- | --- | --- |
| “pain” | During the past 4 weeks, how would you describe the pain you usually had from your knee? | 0 = Severe |
|  |  | 1 = Moderate |
|  |  | 2 = Mild |
|  |  | 3 = Very Mild |
|  |  | 4 = None |
| “night pain” | During the past 4 weeks, have you been troubled by pain from your knee in bed at night? | 0 = Every night |
|  |  | 1 = Most nights |
|  |  | 2 = Some nights |
|  |  | 3 = Only 1 or 2 nights |
|  |  | 4 = No nights |
| “walking” | For how long have you been able to walk before the pain from your knee becomes severe? | 0 = Not at all - pain severe on walking |
|  |  | 1 = Around the house only |
|  |  | 2 = 5-15 minutes |
|  |  | 3 = 16-30 minutes |
|  |  | 4 = No pain/more than 30 minutes |
| “standing” | During the past 4 weeks, after a meal (sat at a table), how painful has it been for you to stand up from a chair because of your knee? | 0 = Unbearable |
|  |  | 1 = Very painful |
|  |  | 2 = Moderately painful |
|  |  | 3 = Slightly painful |
|  |  | 4 = not at all painful |
| “limping” | During the past 4 weeks, have you been limping when walking, because of your knee? | 0 = All of the time |
|  |  | 1 = Most of the time |
|  |  | 2 = Often, not just at first |
|  |  | 3 = Sometimes or just at first |
|  |  | 4 = Rarely/Never |
| “work” | During the past 4 weeks, how much has pain from your knee interfered with your usual work (including housework)? | 0 = Totally |
|  |  | 1 = Greatly |
|  |  | 2 = Moderately |
|  |  | 3 = A little bit |
|  |  | 4 = Not at all |
| “confidence” | During the past 4 weeks, have you felt that your knee might suddenly 'give way' or let you down? | 0 = All of the time |
|  |  | 1 = Most of the time |
|  |  | 2 = Often, not just at first |
|  |  | 3 = Sometimes or just at first |
|  |  | 4 = Rarely/Never |
